# Nutrient Digestibility, Growth Performance, and Blood Indices of Boschveld Chickens Fed Seaweed-Containing Diets

**DOI:** 10.3390/ani10081296

**Published:** 2020-07-29

**Authors:** Lindani Trevor Nhlane, Caven Mguvane Mnisi, Victor Mlambo, Molatelo Junior Madibana

**Affiliations:** 1 Department of Animal Sciences, Faculty of Natural and Agricultural Science, North-West University, P Bag x2046, Mmabatho 2735, South Africa; nhlanelt@gmail.com; 2 Food Security and Safety Niche Area, Faculty of Natural and Agricultural Science, North-West University, P Bag x2046, Mmabatho 2735, South Africa; 3 School of Agricultural Sciences, Faculty of Agriculture and Natural Sciences, University of Mpumalanga, P Bag x11283, Mbombela 1200, South Africa; victor.mlambo@ump.ac.za; 4 Department of Environment, Forestry and Fisheries, Western Cape, Cape Town 8001, South Africa; molateloMA@daff.gov.za

**Keywords:** feed utilisation, growth, haematology, indigenous chickens, seaweeds, serum biochemistry

## Abstract

**Simple Summary:**

Sustainable intensification of indigenous chicken strains is largely constrained by the high cost of feed ingredients, thus limiting the growth of the poultry industry in developing countries. Inexpensive and readily available feed ingredients with nutraceutical properties such as seaweeds can be utilised to address this challenge. Seaweeds have been used in the food, animal, and pharmaceutical industries as a rich source of nutrients and bioactive compounds. Dietary inclusion of seaweeds in chicken diets has the potential to boost growth and enhance product quality. However, their feed value in indigenous chicken diets is largely unknown. This study investigated the effect of graded levels of green seaweed (*Ulva* spp.) meal (SWM) on apparent nutrient digestibility, feed intake, growth performance, and blood parameters of Boschveld indigenous chickens, a South African chicken breed. The inclusion of seaweeds boosted feed intake and overall body weight gain but had no effect on nutrient digestibility and efficiency of feed utilisation. Blood parameters were also not affected, except for red blood cell indicators. It was concluded that green seaweeds have the potential to be included in diets of Boschveld indigenous chickens.

**Abstract:**

Seaweeds possess a wide range of bioactive compounds that can be beneficial in sustainable intensification systems. This study explored the effect of green seaweed (*Ulva* spp.) meal (SWM) on apparent nutrient digestibility and physiological responses of Boschveld indigenous chickens. Two-hundred and seventy-five hens (202.4 ± 6.65 g live-weight; 4-weeks-old) were reared on five isoenergetic and isoproteic diets formulated by adding SWM at 0 (SW0), 20 (SW20), 25 (SW25), 30 (SW30), and 35 g/kg (SW35). Neutral detergent fibre digestibility quadratically responded (R^2^ = 0.244; *p* = 0.042) to SWM levels. No significant dietary influences were observed on apparent nutrient digestibility values. Repeated measures analysis showed significant diet × week interaction effect on weekly feed intake and growth performance. Dietary inclusion of SWM increased (*p* < 0.05) feed intake and overall body weight gain but not feed conversion efficiency. No significant linear and quadratic trends were observed for blood parameters except for basophils (R^2^ = 0.172; *p* = 0.047), which linearly declined with SWM levels. Dietary SWM inclusion only influenced (*p* < 0.05) mean corpuscular volume, mean corpuscular concentration, and mean corpuscular haemoglobin concentration. We concluded that seaweeds have the potential to be used as a feed ingredient for indigenous chickens.

## 1. Introduction

In tropical countries, poultry production is undergoing dramatic changes as large-scale commercial production expands in response to surging demand for poultry meat and eggs [1]. Nearly all rural families in these countries keep a small flock of free-range chickens [2] as a source of dietary essential amino acids, fatty acids, and micronutrients [3]. Despite their low productivity and slower growth rates, indigenous chickens possess desirable characteristics such as thermotolerance, resistance to several diseases, good egg and meat flavour, hard eggshells, and high fertility and hatchability [4]. In order to ensure food and nutrition security of people living in rural settlements, it is important to improve the productivity of indigenous chicken breeds under intensive production conditions. However, sustainable intensification and optimum production requires proper health care and adequate supply of nutrients, which is a major constraint for resource-poor farmers [4]. The high costs of feed ingredients such as maize, soybeans, and feed additives [5] negatively impact on economic sustainability of intensively produced poultry. Therefore, there is a need to identify and evaluate alternative feed ingredients that are not only inexpensive and readily available but also possess growth-boosting and health-promoting properties. Seaweeds or marine macroalgae are candidate nutraceutical plants that can be used in diets of indigenous chickens to provide nutritional and subtherapeutic benefits, which could reduce overreliance on synthetic feed additives that are commonly employed in intensive poultry production systems. 

Seaweeds (*Ulva* spp.) have been used as marine vegetables in Asian and European countries for both human consumption and animal feeding [6]. However, in Africa, seaweeds are mainly used for animal feeding mostly in fish diets [7]. Seaweeds are rich in bioactive compounds with antioxidant, antimicrobial, anti-coagulant, and anti-inflammatory activities [8,9]. In other studies, seaweeds have been used as a source of protein, essential amino acids, vitamins, and minerals [10,11]. Indeed, seaweeds are reported to provide the food or pharmaceutical industries with various ingredients such as pigments, hydrocolloids, vitamins, chelated micro-minerals, and prebiotic substances in the form of polysaccharides and phlorotannins [12]. In addition, seaweeds are used in medicine to treat iodine deficiency and intestinal disorders as hypocholesterolaemic and hypoglycaemic agents [13]. There is potential for their use in poultry production to improve the birds’ immune status and decrease pathogenic microbes in the digestive tract [14]. However, despite their abundancy in some coastal parts of southern Africa, the utility of seaweeds as natural dietary additives on nutrient utilisation and physiological responses in indigenous chicken strains is unknown. This study represents the first ever attempt to use green seaweed in diets of Boschveld indigenous chickens. The objective was to determine apparent nutrient digestibility, feed intake, growth performance, and haematological and serum biochemical parameters of Boschveld indigenous hens fed graded levels of green seaweed meal-containing diets. It was hypothesized that the inclusion of green seaweed meal in Boschveld indigenous chicken diets would not compromise nutrient utilisation, growth performance, and haemato-biochemical parameters of the birds.

## 2. Materials and Methods

The experimental procedures employed in the rearing of the hens conformed to the guidelines for care and use of research animals and were approved by the North-West University Animal Production Sciences Research Ethics Committee (approval no. NWU-00357-19-A5).

### 2.1. Study Site and Ingredient Source

The study was conducted between August and November 2019 at the North-West University, Molelwane Research Farm (25°86′00″ S, 25°64′32″ E) located in the North West province of South Africa. During this time, ambient temperatures ranged from 22 °C to 33 °C. The green seaweed (*Chlorophyceae*; *Ulva* spp.) was collected from an abalone farm, Aquinion in Gansbaai (34°34′58″ S 19°21′8″ E), found in the Western Cape province of South Africa. The seaweed was harvested from a pond and left to drain in an oyster net overnight. The seaweed was then collected in buckets and sun-dried until no water was visible on the leaves at the Marine Research Aquarium of the Department of Environment, Forestry and Fisheries. The seaweed was then oven-dried (60 °C) until constant weight and ground (2-mm, Polymix PX-MFC 90 D) to produce the seaweed meal (SWM). All the other feed ingredients were purchased from Nutroteq (Pretoria, South Africa).

### 2.2. Diet Formulation and Chemical Analyses

A preliminary chemical analyses of SWM was conducted using Association of Official Agricultural Chemists (AOAC) international methods [15] and contained 856.5 g/kg dry matter (DM), 375.2 g/kg DM ash, 175.9 g/kg DM crude protein, 339.3 g/kg DM crude fat, 322.5 g/kg DM neutral detergent fibre, and 171.1 g/kg DM acid detergent fibre. Thereafter, five isoproteic and isoenergetic diets in a mash form (Table 1) were formulated by diluting a commercial grower diet with graded levels of seaweed meal using a feed formulation software. The experimental diets were formulated as follows: (1) SW0 = a commercial grower diet without seaweed meal, (2) SW20 = a commercial grower diet with 2% seaweed meal, (3) SW25 = a commercial grower diet with 2.5% seaweed meal, (4) SW30 = a commercial grower diet with 3% seaweed meal, and (5) SW35 = a commercial grower diet with 3.5% seaweed meal. 

Subsamples of the experimental diets were also analysed for laboratory DM, ash, crude protein, crude fibre, and crude fat using the AOAC international methods [15]. Minerals were analysed using the dry ashing macro and trace minerals method following the guidelines by Agri-laboratory Association of Southern Africa [16]. Metabolisable energy was predicted using models from NIRs SpectraStar XL (Unity Scientific, Emu Plains, Australia).

### 2.3. Growth Performance

A total of 275, three-week-old Boschveld chicks were purchased from Boschveld Ranching (PTY) LTD (Bela-Bela, Limpopo, South Africa). The birds were randomly and evenly allotted to 25 replicate pens (experimental units) measuring 3.5 m length × 1.0 m breadth × 1.85 m height, with each pen holding 11 bird; experiments were replicated five times per dietary treatment. The pens were made of steel wire mesh and stood 20 cm above ground level. Polythene plastics were used as bedding and were regularly replaced. Poltek poultry feeder tubes were used to supply the dietary treatments, and the birds were habituated to the experimental diets for one week. At four weeks of age (202.4 ± 6.65 g live-weight), measurements commenced until 14 weeks of age. Average weekly feed intake (AWFI), average weekly body weight gain (ABWG), and feed conversion efficiency (FCE) were computed as described by Atela et al. [4]. All the experimental pens were checked regularly for sicknesses and mortalities. No mortalities were recorded, thus giving a 100% survival rate. The average temperature (30 °C) and humidity (40%) of the house was regularly monitored using a multi-meter device. Experimental diets and clean water were offered to birds ad libitum, and rearing was conducted under natural lighting (12 h of daylight).

### 2.4. Haematological and Serum Biochemical Parameters

At 14 weeks of age, two chickens from each pen were randomly selected and blood was withdrawn from the brachial vein using sterilised syringes (5 mL) and needles (21 gauge). The blood was immediately transferred into whole blood and sera tubes. Haematological and serum biochemical parameters were analysed using an automated IDEXX LaserCyte Haematology Analyser and an automated IDEXX Catalyst One Chemistry Analyser (IDEXX Laboratories Inc., Westbrook, ME, USA), respectively.

### 2.5. Apparent Nutrient Digestibility Trial

Fifty, Boschveld hens (14 weeks old) from the growth trial were housed in pairs in 25 metabolic cages (0.51 × 0.49 × 0.36 m). The hens were assigned to the same experimental diets (SW0, SW20, SW25, SW30, and SW35) as described in the growth trial. The birds were adapted for three days and thereafter measurements commenced over a period of five days. Samples of feed offered, feed refused, and faeces were collected, pooled, weighed, and processed as described by Manyeula et al. [3]. Apparent digestibility values of dry matter (DMD), organic matter (OMD), crude protein (CPD), neutral detergent fibre (NDFD), and acid detergent fibre (ADFD) were calculated using the following formula:

(1) $$Apparent\;nutrient\;digestibility\;\left(\%\right)=\frac{\left(Nutrient\;intake-Faecal\;nutrients\right)}{Nutrient\;intake}\times 100\;$$

### 2.6. Statistical Analysis

Polynomial contrasts were used to evaluate data for linear and quadratic effects. Response surface regression analysis [17] was applied to estimate the optimum inclusion level of seaweed meal according to the following quadratic model: *y = ax*^2^* + bx + c*, where *y* = response variable; *a* and *b* = coefficients of the quadratic equation; *c* = intercept; *x* = seaweed levels (%); and *−b/*2*a* = *x* value for optimal response. Average weekly feed intake, ABWG, and FCE were analysed using repeated measures procedure of SAS [17] to determine the interaction effect of week and diets. In a completely randomised design, a one-way ANOVA was used to account for dietary effects on apparent nutrient digestibility, feed intake, growth performance, haematology, and serum biochemistry using general linear model procedure of SAS [17]. For all statistical tests, significance was set at *p* < 0.05. Least square means (LSMEANS) were compared using the probability of difference option in the LSMEANS statement of SAS.

## 3. Results

### 3.1. Apparent Nutrient Digestibility

Neither linear nor quadratic effects (*p* > 0.05) were observed for DMD, OMD, CPD, and ADFD in response to SWM levels (Table 2). However, NDFD quadratically responded (*y* = 223.3 (±68.53) + 18.4 (±7.98) *x* − 0.498 (±0.223) *x*^2^; R^2^ = 0.244; *p* = 0.042) to SWM levels. There were no significant dietary influences on DMD, OMD, CPD, NDFD, and ADFD values.

### 3.2. Feed Intake and Growth Performance

Repeated measures analysis showed significant diet × week interaction effects on AWFI, ABWG, and FCE. Table 3 shows that there were linear effects (*p* < 0.05) observed for AWFI in weeks 5, 6, 7, 10, 11, 13, and 14 in response to SWM levels. 

Table 4 indicates that no quadratic trends (*p* > 0.05) were observed for AWFI, except in week 5 where a quadratic response was observed (*y* = 0.015 (±0.004) *x*^2^ − 0.373 (±0.141) *x* + 247.2 (±1.110); R^2^ = 0.203; *p* = 0.001) with SWM levels. There were no significant dietary effects on AWFI in weeks 8, 9, 10, and 12. The control diet SW0 promoted lower (*p* < 0.05) AWFI than did diet SW35 in weeks 5, 7, 11, and 14. In weeks 6 and 13, the control diet SW0 promoted lower AWFI than did diet SW25, but promoted the same (*p* > 0.05) AWFI as diets SW20, SW30, and SW35. Whereas, in week 7, the control diet SW0 promoted the same (*p* > 0.05) AWFI as did diets SW20, SW25, and SW30. In week 11, hens in diets SW25 and SW35 had the highest AWFI compared to those in the control diet SW0. In week 14, diet SW0 had the lowest AWFI (529.5 g/bird) compared to that of diets SW25, SW30, and SW35, which did not differ (*p* > 0.05). Nonetheless, hens fed diets SW0 and SW20 had the same (*p* > 0.05) AWFI for the entire study period.

Table 5 shows that linear effects (*p* > 0.05) were only observed for ABWG in weeks 7 and 14. ABWG linearly increased in week 7 (*y* = 124.9 (±3.898) + 0.169 (±0.496) *x*; R^2^ = 0.334, *p* = 0.007) and week 14 (*y* = 104.5 (±5.474) + 0.708 (±0.697) *x*; R^2^ = 0.203; *p* = 0.021) in response to SWM levels. Significant quadratic trends were only observed for ABWG in week 5 (*y* = 0.028 (±0.009) *x*^2^ − 0.860 (±0.308) *x* + 102.0 (±2.422); R^2^ = 0.316; *p* = 0.005) and week 13 (*y* = 56.8 (±8.90) + 3.50 (±1.133) *x* − 0.090 (±0.033) *x*^2^; R^2^ = 0.247; *p* = 0.012). There were significant dietary effects on ABWG in weeks 5 and 13. In week 5, diet SW35 (107.3 g/bird) promoted the highest ABWG compared with that of diets SW20 and SW30, but promoted similar (*p* > 0.05) ABWG as did diets SW0 and SW25. In week 13, hens in the control diet SW0 (56.71 g/bird) had lower ABWG than those in diet SW25 (96.7 g/bird). Nonetheless, hens in the control diet SW0 had the same (*p* > 0.05) ABWG as those fed diets SW20, SW30, and SW35 for the entire duration of the study. 

Table 6 shows that FCE linearly declined in week 6 (*y* = 0.410 (±0.0077) − 0.0004 (±0.0010) *x*; R^2^ = 0.364; *p* = 0.002) and week 10 (*y* = 0.245 (±0.0061) − 0.001 (±0.0008) *x*; R^2^ = 0.397; *p* = 0.001) in response to SWM levels. There were quadratic trends (*p* < 0.05) for FCE only in week 5 (*y* = 0.00009 (±0.00003) *x*^2^ − 0.003 (±0.001) *x* + 0.412 (±0.009); R^2^ = 0.243; *p* = 0.017) and week 13 (*y* = 0.118 (±0.0141) + 0.005 (±0.0018) *x* − 0.0001 (±0.00005) *x*^2^; R^2^ = 0.269, *p* = 0.010) in response to SWM level. There were significant dietary effects on FCE in weeks 5, 6, 10, and 11. In week 5, hens fed the control diet SW0 had similar (*p* > 0.05) FCE as those fed the SWM-containing diets. Diet SW20 (0.388) promoted the least FCE compared with those of diet SW35 (0.426). In week 6, the control diet SW0 (0.410) promoted the highest (*p* > 0.05) FCE compared to those of diets SW30 and SW35 but had similar FCE as did diets SW20 and SW25. In week 10, hens fed the control diet SW0 (0.410) had the highest FCE compared with those fed diets SW25, SW30, and SW35, which did not differ (*p* > 0.05). In week 11, hens fed the control diet SW0 had higher (*p* < 0.05) FCE than those in diet SW25. However, the control diet SW0 promoted the same (*p* > 0.05) FCE as diets SW20, SW30, and SW35.

### 3.3. Haemato-Biochemistry

Neither linear nor quadratic responses (*p* > 0.05) were observed for haematological parameters (Table 7), except for basophils, which linearly decreased (*y* = 2.06 (±0.732) − 0.120 (±0.095) *x*; R^2^ = 0.172, *p* = 0.047) in response to SWM levels. 

There were no dietary effects (*p* > 0.05) on haematological parameters with the exception of MCV, MCH, and MCHC. Hens in diet SW20 had the lowest MCV (76.60 fL) compared with those in diet SW25 (84.66 fL). Hens fed diet SW30 had the highest MCH (52.26 pg) compared with those in diets SW20 and SW35, which did not differ (*p* > 0.05). Diet SW30 (0.645 g/dL) promoted the highest MCHC compared with those of diets SW20, SW25, and SW35, which were similar (*p* > 0.05). Nonetheless, the SWM-containing diets promoted the same (*p* > 0.05) MCV, MCH, and MCHC as the control diet SW0.

There were no significant linear and quadratic trends for serum biochemical parameters in response to SWM levels (Table 8). Similarly, no dietary effects (*p* > 0.05) were observed on all serum biochemical indices.

## 4. Discussion

### 4.1. Apparent Nutrient Digestibility

Nutrient digestibility is the extent to which feed nutrients are absorbed as they pass through the bird’s digestive tract. In this study, the inclusion of SWM in Boschveld indigenous hens did not alter dry matter, organic matter, crude protein, and neutral and acid detergent fibre digestibility values of the birds. These findings were consistent with those of Burgin [14] who found that the inclusion of green seaweed from 2% to 4% in broiler diets did not compromise nutrient availability. According to Jimenez-Escrig and Sanchez-Muniz [18], seaweeds contain between 250 and 750 g/kg DM of dietary fibre, which could reduce nutrient digestibility. Results from this study showed that a significant quadratic trend was observed for NDFD in response to increasing dietary levels of SWM, which indicates that the presence of polysaccharides (fucoidan, laminarin, alginate, and cellulose) in the seaweed [18,19] may reduce its utilisation at higher inclusion rates. Indeed, non-starch polysaccharides such as cellulose, pectins, arabinoxylans, and *β*-glucans are known to suppress nutrient digestibility in poultry [20]. 

### 4.2. Feed Intake and Growth Performance

Seaweeds have nutraceutical properties that can be exploited to improve the performance of chickens [21,22]. Repeated measures analysis revealed significant week × diet interaction effects on feed intake, body weight gain, and feed conversion efficiency, which indicates that the capacity of the hens to utilise seaweed-containing diets depended on their age. Linear effects were observed for feed intake in weeks 5, 6, 7, 10, 11, 13, and 14 in response to SWM levels, which could be due to the presence of non-starch polysaccharides present in the seaweed [18]. Indeed, the crude fibre content of the experimental diets increased with increasing levels of dietary SWM. Fibre is known to interfere with the utilisation of nutrients, thus, the extra dietary fibre in the SWM-containing diets might have caused low blood sugar levels, prompting the birds to consume more feed. This could also explain why birds in the control diet had lower feed intake than those on diet SW25 in weeks 6 and 13, and those on diet SW35 in weeks 5, 7, 11, and 14. A negative quadratic response was observed for feed intake in week 5 in response to dietary SWM levels, indicating that the young birds responded by increasing feed intake as a way to cater for nutrient dilution. However, a study conducted by El-Deek and Brikka [23] revealed that SWM can be included in poultry diets at rates of 5 to 15%, depending on the species of algae and animal age. In other studies, feed intake was adversely affected by the inclusion of SWM at 3% [21,24].

Seaweeds have been reported to have growth-stimulating effects [25], which could explain the significant linear increase in weeks 7 and 14 and the negative quadratic response in week 5 on body weight gain of the hens. Hens in the control diet had the same overall body weight gain as those fed 35 g/kg SWM, which shows that the highest inclusion level of SWM in this current study did not suppress body weight gain. These findings were consistent with those of Abudabos et al. [24] and El-Deek et al. [26], who reported that seaweed inclusion has no significant effect on body weight gain of broilers. Choi et al. [27] showed that supplementation of seaweed by products at 5 g/kg has positive effects on FCE. However, results from this study showed that the inclusion of SWM depressed the FCE of the birds, which could be attributed to the high dietary fibre levels of the seaweed.

### 4.3. Haematological and Serum Biochemical Indices

Haematological indices serve as indicators of the physiological state of farm animals [28] and are helpful in monitoring feed toxicity, especially when a new feed ingredient is introduced to an animal [29]. Results from this study show that no significant linear or quadratic trends were observed for all haematological parameters with the exception for basophils, which linearly decreased in response to SWM levels. Low basophils, known as basopenia, is usually caused by an infection or an allergic reaction. Thus, the low basophils observed in hens supplemented with SWM suggests that the birds could have had an allergic reaction to dietary seaweeds. However, the basophils values fell within the normal range reported for healthy birds [30]. In addition, seaweed inclusion had significant influence on red blood cell indicators (MCV, MCH, and MCHC) of the birds. However, the MCV and MCH values fell within the normal ranges reported for healthy birds, whereas the MCHC values were slightly below the reference values [31]. The MCHC is a diagnostic indicator for anaemia and serves as a useful index of the capacity of the bone marrow to produce red blood cells [32], whereas MCH is an indicator of haemoglobin in the red blood cells. In this study, birds on diet SW30 had the highest MCH and MCHC values, which could suggest that the birds may be more efficient in performing respiratory functions, as observed by Soetan et al. [33] and Abdulazeez et al. [34]. Nonetheless, the MCV, MCH, and MCHC values were similar to those observed in birds on the control diet.

The serum biochemical parameters of the hens were not influenced by dietary SWM inclusion, which indicates that the intake of seaweeds has no negative post-ingestive feedback. Indeed, the liver enzymes (ALT, ALKP, and GGT), which are important to determine proper liver functioning [35], were not altered, suggesting that seaweeds have the potential to be used in diets of Boschveld indigenous chickens. According to Jiwuba et al. [36] serum albumin concentration reflects liver functioning, thus, the higher albumin reported in this study compared to the findings by Albokhadaim [37] and Sugiharto et al. [38] indicates a better liver functioning of the birds in response to dietary seaweed. Furthermore, the lack of dietary differences reported for total protein, urea, and creatinine implies that seaweed-containing diets promote similar renal function as the control diet. Likewise, the similarities in glucose and amylase concentrations among the birds signify normal fat metabolism, which shows that the inclusion of seaweed has no detrimental effects on the health status of hens.

## 5. Conclusions

The inclusion of green seaweed meal significantly increased feed intake and overall body weight gain but did not improve apparent nutrient digestibility and feed conversion efficiency of Boschveld indigenous hens. Blood parameters were similar to the control diet, suggesting that the use of seaweed did not compromise the well-being of the birds. It can be concluded that seaweed meal does not compromise the performance and health status of the birds. 

## Figures and Tables

**Table 1:** Ingredients and chemical composition (g/kg as-fed basis, unless stated otherwise) of diets containing seaweed meal.

	Diets ^1^				
	SW0	SW20	SW25	SW30	SW35
Ingredients					
Seaweed meal	0.0	20.0	25.0	30.0	35.0
Yellow maize 8%	630.3	643.7	647.3	648.1	648.8
Extruded full-fat soya	120.0	81.1	61.5	46.6	31.9
Soya oilcake 47%	176.6	192.8	203.0	207.5	211.9
Sunflower oilcake 36%	30.0	30.0	31.6	36.6	41.5
Limestone	11.9	9.7	9.1	8.5	7.9
Monocalcium phosphate	7.8	8.1	8.1	8.2	8.3
Fine salt	2.6	0.6	0.1	0.0	0.0
Sodium bicarbonate	1.5	1.5	1.5	1.5	1.5
DL methionine	2.8	2.9	2.9	2.9	2.9
_L_-Threonine	0.7	0.8	0.9	0.9	1.0
Lysine HCL	2.7	3.0	3.1	3.3	3.4
Crude soya oil mixer	7.2	0.0	0.0	0.0	0.0
Lignobond	2.5	2.5	2.5	2.5	2.5
Broiler grower meal	2.5	2.5	2.5	2.5	2.5
AxtraPhy10000	0.1	0.1	0.1	0.1	0.1
Salinomycin 12%	0.5	0.5	0.5	0.5	0.5
Zinc Bacitracin	0.3	0.3	0.3	0.3	0.3
Chemical composition					
Dry matter	884.8	882.5	882.1	881.8	881.6
Metabolisable energy (MJ/kg)	12.9	12.9	12.9	12.9	12.9
Crude protein	192.2	192.2	192.2	192.2	192.2
Crude fat	56.2	43.4	40.2	37.9	35.5
Crude fibre	35.5	44.0	46.2	49.1	51.9
Ash	25.1	30.9	32.4	33.9	35.4
Calcium	8.4	8.4	8.4	8.4	8.4
Chloride	2.4	2.7	2.8	3.2	3.5
Sodium	1.8	1.8	1.8	2.0	2.2
Phosphorus	5.5	5.5	5.5	5.5	5.5

^1^ Diets: SW0 = commercial grower diet without seaweed meal; SW20 = commercial grower diet with seaweed meal at a rate of 2%; SW25 = commercial grower diet with seaweed meal at a rate of 2.5%; SW30 = commercial grower diet with seaweed meal at a rate of 3%; SW35 = commercial grower diet with seaweed meal at a rate of 3.5%.

**Table 2:** Apparent nutrient digestibility (g/kg dry matter (DM), unless stated otherwise) of Boschveld indigenous hens fed seaweed-containing diets.

Parameters ^2^	Diets ^1^					SEM ^3^	*p*-Value	
	SW0	SW20	SW25	SW30	SW35		Linear	Quadratic
DMD	588.3	633.9	642.2	630.5	650.5	37.99	0.266	0.297
OMD	532.7	582.5	589.7	570.0	591.6	38.16	0.304	0.181
CPD	230.4	215.1	337.8	337.2	348.0	50.52	0.066	0.417
NDFD	212.2	294.2	430.4	309.4	277.0	59.76	0.547	0.042
ADFD	154.0	139.0	322.4	239.7	173.3	46.36	0.420	0.455

^1^ Diets: SW0 = commercial grower diet without seaweed meal; SW20 = commercial grower diet with seaweed meal at a rate of 2%; SW25 = commercial grower diet with seaweed meal at a rate of 2.5%; SW30 = commercial grower diet with seaweed meal at a rate of 3%; SW35 = commercial grower diet with seaweed meal at a rate of 3.5%. ^2^ Parameters: DMD = dry matter digestibility; OMD = organic matter digestibility; CPD = crude protein digestibility; NDFD = neutral detergent fibre digestibility; ADFD = acid detergent fibre digestibility. ^3^ SEM = standard error of the mean.

**Table 3:** Regression equations for average weekly feed intake of Boschveld indigenous hens in response to graded levels of seaweed meal.

Week	Linear	R^2^	*p*-Value
5	*y* = 247.2 (±1.110) − 0.373 (±0.141) *x*	0.312	0.007
6	*y* = 278.4 (±5.504) + 0.631 (±0.700) *x*	0.251	0.015
7	*y* = 350.3 (±7.842) − 0.714 (±0.998) *x*	0.234	0.014
10	*y* = 460.9 (±14.76) + 2.743 (±1.879) *x*	0.283	0.008
11	*y* = 441.3 (±14.62) + 2.641 (±1.862) *x*	0.336	0.008
13	*y* = 478.3 (±21.90) + 4.366 (±2.788) *x*	0.209	0.026
14	*y* = 526.6 (±23.66) + 4.084 (±3.012) *x*	0.409	0.001

**Table 4:** Average weekly feed intake (g/bird) of Boschveld indigenous hens fed diets containing seaweed meal.

Week	Diets ^1^					SEM ^2^	*p*-Value	
	SW0	SW20	SW25	SW30	SW35		Linear	Quadratic
5	247.3 ^a^	245.2 ^a^	247.5 ^ab^	249.5 ^ab^	252.1 ^b^	1.177	0.007	0.001
6	278.8 ^a^	284.0 ^ab^	301.8 ^b^	290.9 ^ab^	297.1 ^ab^	5.015	0.015	0.901
7	351.1 ^ab^	343.8 ^a^	375.0 ^bc^	365.1 ^abc^	379.2 ^c^	6.967	0.014	0.131
8	407.8	393.0	421.9	405.1	418.7	9.029	0.459	0.315
9	446.9	441.1	496.7	450.9	471.9	18.224	0.302	0.986
10	461.6	493.3	520.2	509.8	513.6	14.770	0.008	0.534
11	442.7 ^a^	465.3 ^ab^	517.2 ^b^	496.0 ^ab^	499.7 ^b^	13.389	0.008	0.534
12	467.6	459.1	525.6	500.6	508.2	17.118	0.070	0.681
13	479.4 ^a^	516.4 ^ab^	579.8 ^b^	521.2 ^ab^	546.2 ^ab^	19.696	0.026	0.379
14	529.5 ^a^	568.8 ^ab^	646.7 ^b^	625.1 ^b^	630.4 ^b^	23.160	0.001	0.809

^1^ Diets: SW0 = commercial grower diet without seaweed meal; SW20 = commercial grower diet with seaweed meal at a rate of 2%; SW25 = commercial grower diet with seaweed meal at a rate of 2.5%; SW30 = commercial grower diet with seaweed meal at a rate of 3%; SW35 = commercial grower diet with seaweed meal at a rate of 3.5%. ^2^ SEM = standard error of the mean. ^a,b,c^ In a row, means with different superscripts significantly differ at *p* < 0.05.

**Table 5:** Average weekly body weight gain (g/bird) of Boschveld indigenous hens fed diets containing seaweed meal.

Week	Diets ^1^					SEM ^2^	*p*-Value	
	SW0	SW20	SW25	SW30	SW35		Linear	Quadratic
5	102.0 ^ab^	95.17 ^a^	100.9 ^ab^	98.03 ^a^	107.3 ^b^	2.28	0.443	0.005
6	114.1	112.8	117.5	108.8	112.0	2.51	0.506	0.492
7	125.0	127.2	134.8	134.4	141.4	3.93	0.007	0.217
8	117.0	114.3	106.5	104.7	114.1	6.41	0.502	0.439
9	125.0	125.7	131.5	119.2	124.7	3.10	0.797	0.364
10	112.9	112.0	113.7	111.0	111.0	2.99	0.658	0.762
11	100.6	109.7	96.54	110.4	108.5	4.61	0.363	0.844
12	77.6	54.5	74.5	70.6	70.1	7.31	0.568	0.185
13	56.7 ^a^	88.0 ^ab^	96.7 ^b^	71.1 ^ab^	72.0 ^ab^	8.62	0.144	0.012
14	104.4	105.3	109.4	113.4	124.8	5.76	0.021	0.082

^1^ Diets: SW0 = commercial grower diet without seaweed meal; SW20 = commercial grower diet with seaweed meal at a rate of 2%; SW25 = commercial grower diet with seaweed meal at a rate of 2.5%; SW30 = commercial grower diet with seaweed meal at a rate of 3%; SW35 = commercial grower diet with seaweed meal at a rate of 3.5%. ^2^ SEM = standard error of the mean. ^a,b^ In a row, means with different superscripts significantly differ at *p* < 0.05.

**Table 6:** Average weekly feed conversion efficiency of Boschveld indigenous hens fed diets containing seaweed meal.

Week	Diets ^1^					SEM ^2^	*p*-Value	
	SW0	SW20	SW25	SW30	SW35		Linear	Quadratic
5	0.412 ^ab^	0.388 ^a^	0.408 ^ab^	0.393 ^ab^	0.426 ^b^	0.009	0.816	0.017
6	0.410 ^b^	0.397 ^ab^	0.389 ^ab^	0.374 ^a^	0.377 ^a^	0.008	0.002	0.535
7	0.356	0.370	0.359	0.368	0.373	0.007	0.108	0.923
8	0.288	0.291	0.253	0.259	0.273	0.017	0.361	0.887
9	0.280	0.285	0.266	0.266	0.265	0.008	0.081	0.372
10	0.245 ^b^	0.228 ^ab^	0.218 ^a^	0.219 ^a^	0.216 ^a^	0.006	0.001	0.698
11	0.228 ^b^	0.236 ^b^	0.186 ^a^	0.223 ^ab^	0.218 ^ab^	0.010	0.301	0.715
12	0.165	0.120	0.140	0.113	0.137	0.018	0.120	0.143
13	0.118	0.169	0.166	0.136	0.132	0.014	0.350	0.010
14	0.198	0.185	0.170	0.182	0.198	0.009	0.479	0.057

^1^ Diets: SW0 = commercial grower diet without seaweed meal; SW20 = commercial grower diet with seaweed meal at a rate of 2%; SW25 = commercial grower diet with seaweed meal at a rate of 2.5%; SW30 = commercial grower diet with seaweed meal at a rate of 3%; SW35 = commercial grower diet with seaweed meal at a rate of 3.5%. ^2^ SEM = standard error of the mean. ^a,b^ In a row, means with different superscripts significantly differ at *p* < 0.05.

**Table 7:** Haematological parameters of Boschveld indigenous hens fed diets containing seaweed meal.

Parameters ^2^	Diets ^1^					SEM ^3^	*p*-Value	
	SW0	SW20	SW25	SW30	SW35		Linear	Quadratic
RBC (×10^12^/L)	2.23	2.40	2.41	2.08	2.54	0.151	0.651	0.881
Haematocrit (%)	18.68	18.40	20.37	16.76	20.24	1.173	0.946	0.965
Haemoglobin (g/dL)	9.76	9.23	8.96	9.58	9.99	0.557	0.916	0.213
MCV (fL)	81.83 ^ab^	76.60 ^a^	84.66 ^b^	81.02 ^ab^	79.59 ^ab^	1.516	0.722	0.768
MCH (pg)	45.30 ^ab^	39.01 ^a^	41.99 ^ab^	52.26 ^b^	39.47 ^a^	2.660	0.977	0.487
MCHC (g/dL)	0.55 ^ab^	0.51 ^a^	0.50 ^a^	0.65 ^b^	0.50 ^a^	0.033	0.926	0.559
RDW (%)	25.28	26.48	24.88	26.13	25.79	0.491	0.501	0.679
Neutrophils (×10^9^/L)	22.41	62.08	13.79	14.28	8.00	15.841	0.480	0.094
Lymphocytes (×10^9^/L)	138.4	167.4	101.1	134.1	61.18	47.90	0.439	0.370
Monocytes (×10^9^/L)	38.40	17.34	21.24	50.84	15.17	11.55	0.625	0.573
Eosinophils (×10^9^/L)	1.33	1.68	1.08	2.17	0.93	0.331	0.855	0.500
Basophils (×10^9^/L)	2.06	0.39	0.16	0.31	0.07	0.750	0.047	0.509
Platelets (K/µL)	95.50	109.3	117.6	133.0	111.4	13.97	0.146	0.739
MPV (fL)	7.54	6.86	10.77	5.22	7.84	1.258	0.892	0.588

^1^ Diets: SW0 = commercial grower diet without seaweed meal; SW20 = commercial grower diet with seaweed meal at a rate of 2%; SW25 = commercial grower diet with seaweed meal at a rate of 2.5%; SW30 = commercial grower diet with seaweed meal at a rate of 3%; SW35 = commercial grower diet with seaweed meal at a rate of 3.5%. ^2^ Parameters: RBC = red blood cell count; MCV = mean corpuscular volume; MCH = mean corpuscular haemoglobin; MCHC = mean corpuscular haemoglobin concentration; RDW = red cell distribution width; MPV = mean platelet volume. ^3^ SEM = standard error of the mean. ^a,b^ In a row, means with different superscripts significantly differ at *p* < 0.05.

**Table 8:** Serum biochemical parameters of Boschveld indigenous hens fed diets containing seaweed meal.

Parameters ^2^	Diets ^1^					SEM ^3^	*p*-Value	
	SW0	SW20	SW25	SW30	SW35		Linear	Quadratic
Glucose (mmol/L)	1.95	1.90	4.67	3.75	3.29	0.960	0.194	0.867
SDMA (µg/dL)	55.00	67.20	57.30	61.10	67.60	10.612	0.985	0.736
Urea (mmol/L)	0.84	0.89	0.69	0.84	0.88	0.051	0.793	0.126
Phosphorus (mmol/L)	4.33	5.00	4.44	3.94	4.51	0.337	0.628	0.554
Calcium (mmol/L)	2.05	2.74	2.11	2.28	2.40	0.271	0.755	0.517
Total protein (g/L)	69.20	89.40	76.80	65.23	76.15	8.577	0.935	0.601
Albumin (g/L)	25.20	31.32	28.00	26.90	36.50	3.484	0.312	0.236
ALT (U/L)	43.32	57.44	51.40	55.40	48.15	7.411	0.607	0.679
ALKP (U/L)	259.8	254.0	214.6	234.8	199.5	30.364	0.065	0.447
Bilirubin (µmol/L)	25.10	39.20	32.50	22.70	24.25	7.350	0.769	0.457
Amylase (U/L)	900.3	912.7	890.5	922.9	820.9	100.35	0.898	0.479
Creatinine (µmol/L)	22.70	38.30	16.80	16.00	34.60	7.378	0.927	0.151
Globulin (g/L)	44.60	52.23	52.90	47.50	48.50	3.559	0.577	0.213
ALB/GLOB	0.60	0.57	0.48	0.46	0.54	0.044	0.052	0.343
GGT (U/L)	15.75	7.48	17.30	18.88	10.25	3.314	0.701	0.357

^1^ Diets: SW0 = commercial grower diet without seaweed meal; SW20 = commercial grower diet with seaweed meal at a rate of 2%; SW25 = commercial grower diet with seaweed meal at a rate of 2.5%; SW30 = commercial grower diet with seaweed meal at a rate of 3%; SW35 = commercial grower diet with seaweed meal at a rate of 3.5%. ^2^ Parameters: SDMA = symmetric dimethylarginine; ALB/GLOB = albumin to globulin ratio; ALT = alanine aminotransferase; ALKP = alkaline phosphatase; GGT = gamma-glutamyl transferase. ^3^ SEM = standard error of the mean.

## References

[1] Dessie T., Dana N., Ayalew W., Hanotte O. (2012). Current state of knowledge on indigenous chicken genetic resources of the tropics: Domestication, distribution and documentation of information on the genetic resources. Worlds Poult. Sci. J..

[2] Riise J.C., Permin A., Kryger O.N. (2004). Strategies for developing family poultry production at village level. Experiences from West Africa and Asia. Worlds Poult. Sci. J..

[3] Manyeula F., Mlambo V., Marume U., Sebola N.A. (2019). Nutrient digestibility, haemo-biochemical parameters and growth performance of an indigenous chicken strain fed canola meal–containing diets. Trop. Anim. Health Prod..

[4] Atela J., Mlambo V., Mnisi C.M. (2019). A multi-strain probiotic administered via drinking water enhances feed conversion efficiency and meat quality traits in indigenous chickens. Anim. Nutr..

[5] Schmit T.M., Verteramo L., Tomek W.G. (2009). Implications of growing biofuels demands on Northeast livestock feed costs. Agric. Resour. Econ. Rev..

[6] Fleurence J., LeCoeur C., Mabeau S., Maurice M., Landrein A. (1995). Comparison of different extractive procedures for proteins from the edible seaweeds *Ulva rigida* and *Ulva rotundata*. J. Appl. Phycol..

[7] Troell M., Joyce A., Chopin T., Neori A., Buschmann A.H., Fang J.G. (2009). Ecological engineering in aquaculture-potential for integrated multi-trophic aquaculture (IMTA) in marine offshore systems. Aquaculture.

[8] Yuan Y.V., Walsh N.A. (2006). Antioxidant and antiproliferative activities of extracts from a variety of edible seaweeds. Food Chem. Toxicol..

[9] Chandini S.K., Ganesan P., Bhaskar N. (2008). In vitro antioxidant activities of three selected brown seaweeds of India. Food Chem..

[10] Kumar V., Kaladharan P. (2007). Amino acids in the seaweeds as an alternative source of protein for animal feed. J. Mar. Biol. Assoc. India.

[11] Lange K.W., Hauser J., Nakamura Y., Kanaya S. (2015). Dietary seaweeds and obesity. Food Sci. Hum. Wellness.

[12] Evans F.D., Critchley A.T. (2014). Seaweeds for animal production use. J. Appl. Phycol..

[13] Makkar H.P.S., Tranb G., Heuzé V., Giger-Reverdinc S., Lessiree M., Lebas F., Ankers P. (2016). Seaweeds for livestock diets: A review. Anim. Feed. Sci. Technol..

[14] Burgin R. (2016). Benefits of Seaweed in Poultry Diets. Poultry World. https://www.poultryworld.net/Nutrition/Articles/2016/2/Benefits-of-seaweed-in-poultry-diets-2761921W.

[15] Association of Official Analytical Chemists (2005). Official Methods of Analysis of Association of Official Analytical Chemists International.

[16] Agri Laboratory Association of Southern Africa (1998). Feed and Plant. Analysis Methods.

[17] Statistical Analysis System Institute Inc. (2010). Users Guide.

[18] Jimenez-Escrig A., Sanchez-Muniz F.J. (2000). Dietary fibre from edible seaweeds: Chemical structure, physicochemical properties and effects on cholesterol metabolism. Nutr. Res..

[19] Lahaye M., Jegou D. (1993). Chemical and physical–chemical characteristics of dietary fibres from *Ulva lactuca* (L.) Thuret and *Enteromorpha compressa* (L.) Grev. J. Appl. Phycol..

[20] Madzimure J., Muchapa L., Gwiriri L., Bakare A.G., Masaka L. (2017). Growth performance of broilers fed on sprouted-roasted guar bean (*Cyamopsis tetragonoloba*) based diets. Trop. Anim. Health Prod..

[21] Ventura M.R., Castanon J.I.R., McNab J.M. (1994). Nutritional value of seaweed (*Ulva rigida*) for poultry. Anim. Feed Sci. Technol..

[22] Fleurence J. (1999). Seaweed proteins: Biochemical, nutritional aspects and potential uses. Trends Food Sci. Technol..

[23] El-Deek A.A., Brikka M.A. (2009). Nutritional and biological evaluation of marine seaweed as a feedstuff and as a pellet binder in poultry diet. Int. J. Poult. Sci..

[24] Abudabos A.M., Okab A.B., Aljumaah R.S., Samara E.M., Abdoun K.A., Al-Haidary A.A. (2013). Nutritional value of green seaweed (*Ulva lactuca*) for broiler chickens. Ital. J. Anim. Sci..

[25] Mooney P.A., Van Staden J. (1986). Algae and cytokinins. J. Plant Physiol..

[26] El-Deek A.A., Asar M., Safaa Hamdy M.A., Kosba M.A., Osman M. (1987). Nutritional value of marine seaweed in broiler diets. J. Agric. Sci..

[27] Choi Y.J., Lee S.R., Oh J.W. (2014). Effects of dietary fermented seaweed and seaweed fusiforme on growth performance, carcass parameters and immunoglobulin concentration in broiler chicks. Asian Australas. J. Anim. Sci..

[28] Chowdhury S.R., Smith T.K., Boermans H.J., Woodward B. (2005). Effects of feed-borne Fusarium mycotoxins on hematology and immunology of laying hens. Poult. Sci..

[29] Jiwuba P.C., Onunwa E.C. (2018). Dietary effect of velvet bean (*Mucuna utilis*) leaf meal on haematology and serum biochemistry of broiler finisher birds. Sustain. Food Prod..

[30] Reece O.W. (2009). Functional Anatomy and Physiology of Domestic Animals.

[31] Aengwanic W., Chinrasri O., Simaraks S. (2004). Hematological, electrolyte and serum biochemical values of the Thai indigenous chickens (*Gallus domesticus*) in northeastern, Thailand. Songklanakarin J. Sci. Technol..

[32] Etim N.N., Enyenihi G.E., Williams M.E., Udo M.D., Offiong E.E.A. (2014). Haematological parameters: Indicators of the physiological status of farm animals. Brit. J. Sci..

[33] Soetan K.O., Akinrinde A.S., Ajibade T.O. Preliminary studies on the haematological parameters of cockerels fed raw and processed guinea corn (*Sorghum bicolor*). Proceedings of the 38th Annual Conference of Nigerian Society for Animal Production.

[34] Abdulazeez H., Adamu S.B., Igwebuike J.U., Gwayo G.J., Muhammad A.I. (2016). Haematology and serum biochemistry of broiler chickens fed graded levels of Baobab (*Adansonia digitata* L.) seed meal. J. Agri. Vet. Sci..

[35] Ambrosy A.P., Dunn T.P., Heidenreich P.A. (2015). Effect of minor liver function test abnormalities and values within the normal range on survival in heart failure. Am. J. Cardiol..

[36] Jiwuba P.C., Ugwu D.O., Kadurumba O.E., Dauda E. (2016). Haematological and serum biochemical indices of weaner rabbits fed varying levels of dried *Gmelina arborea* leaf meal. Int. Blood Res. Rev..

[37] Albokhadaim I. (2012). Hematological and some biochemical values of indigenous chickens in Al-Ahsa, Saudi Arabia during rainy season. Asian J. Poult. Sci..

[38] Sugiharto S., Yudiarti T., Isroli I., Widiastuti E. (2018). Effect of feeding duration of *Spirulina platensis* on growth performance, haematological parameters, intestinal microbial population and carcass traits of broiler chicks. S. Afr. J. Anim. Sci..

